# Chromosome Segregation Impacts on Cell Growth and Division Site Selection in *Corynebacterium glutamicum*

**DOI:** 10.1371/journal.pone.0055078

**Published:** 2013-02-06

**Authors:** Catriona Donovan, Astrid Schauss, Reinhard Krämer, Marc Bramkamp

**Affiliations:** 1 Department of Biology I, Ludwig-Maximilians-University Munich, Planegg-Martinsried, Germany; 2 Institute for Biochemistry, University of Cologne, Cologne, Germany; 3 Cologne Excellence Cluster on Cellular Stress Response in Aging-Associated Diseases (CECAD), Cologne, Germany; University of Groningen, Groningen Institute for Biomolecular Sciences and Biotechnology, The Netherlands

## Abstract

Spatial and temporal regulation of bacterial cell division is imperative for the production of viable offspring. In many rod-shaped bacteria, regulatory systems such as the Min system and nucleoid occlusion ensure the high fidelity of midcell divisome positioning. However, regulation of division site selection in bacteria lacking recognizable Min and nucleoid occlusion remains less well understood. Here, we describe one such rod-shaped organism, *Corynebacterium glutamicum*, which does not always place the division septum precisely at midcell. Here we now show at single cell level that cell growth and division site selection are spatially and temporally regulated by chromosome segregation. Mutants defective in chromosome segregation have more variable cell growth and aberrant placement of the division site. In these mutants, division septa constrict over and often guillotine the nucleoid, leading to nonviable, DNA-free cells. Our results suggest that chromosome segregation or some nucleoid associated factor influences growth and division site selection in *C. glutamicum*. Understanding growth and regulation of *C. glutamicum* cells will also be of importance to develop strains for industrial production of biomolecules, such as amino acids.

## Introduction

Many rod-shaped bacteria divide precisely at midcell, generating two equally sized and genetically identical daughter cells. Division site selection is controlled by regulating the positioning of the primary bacterial cell division protein, FtsZ. This tubulin homologue polymerizes at midcell forming a ring-like structure known as the Z-ring, which subsequently primes the midcell for assembly of the division machinery complex [1], [2]. Spatial regulators, such as the Min system and nucleoid occlusion, facilitate midcell localization of the Z-ring and, subsequently, the divisome [3]. The effector proteins of nucleoid occlusion, Noc in *B. subtilis* and SlmA in *E. coli*, are DNA-binding proteins that prevent Z-ring formation over the nucleoid [4], [5], [6]. The Min system prevents aberrant divisions close to the cell poles [7], [8]. In *E. coli*, the Min system oscillates pole to pole, thereby setting up a gradient of the FtsZ inhibitor, MinC, which is lowest at midcell [3], [9], [10], [11], [12]. The *B. subtilis* Min system does not oscillate and has been recently shown to be important for disassembly of the divisome machinery and prevents new rounds of cytokinesis occurring close to the original cell division site [13], [14].

Assembly of the divisome must be temporally coordinated with chromosome replication and segregation to ensure maintenance of genomic integrity. Bacterial DNA segregation is often mediated by the tripartite partitioning system encoded by the *par* locus. Two *trans*-acting proteins, encoded in an operon (*parA* and *parB*, respectively), and *cis*-acting “centromere-like” elements, found scattered around the origin of replication (*oriC*), constitute the Par segregation machinery [15], [16], [17]. The centromere-binding protein (ParB) binds the centromere-like element (*parS*) forming a nucleoprotein complex [18]. Dynamic localization of the Walker type P-loop ATPase (ParA), has been suggested to mediate segregation of the ParB-*parS* nucleoprotein complexes [19].

In the absence of recognizable homologues of the Min system and nucleoid occlusion (Noc or SlmA), bacteria have developed alternative strategies to spatially and temporally regulate cell division. A well-studied example is *Caulobacter crescentus*, which strictly synchronises chromosome segregation with cell division [20], . In the predivision cell, the negative regulator of Z-ring polymerization, MipZ, forms a gradient extending from the origin tethered pole towards the midcell region [22]. ParB is imperative in maintenance of MipZ gradients [22], . After replication onset and as the chromosome segregates, MipZ travels with the segregating ParB bound origin. At the opposite cell pole, MipZ displaces FtsZ, restricting Z-ring polymerization to the midcell region. Thus, in *C. crescentus* null deletion mutants of the Par system are lethal [24].

Members of the Actinobacteria phylum, such as *Corynebacterium glutamicum*, not only lack homologues of Min and nucleoid occlusion, but also other positive and negative regulators of FtsZ, such as ErzA, FtsA, ZapA or ZipA [25]. Interestingly, the placement of the division septum in this rod-shaped organism is relatively flexible and is not necessarily always positioned precisely at midcell. Previously, we found that the ParAB system works as chromosome partitioning system [26]. Here, cell growth and division site selection in *C. glutamicum* was analyzed at the single cell level. By contrast to wild type cells, in the absence of an active chromosome segregation machinery, disorganised chromosomes additionally impacts on division site selection and growth.

## Results

### Growth of *C. glutamicum* is Regulated by a Size-based Mechanism

Conventionally, growth characterisation of microbial cells involved analysis of cell populations as a whole, which, although overall informative, neglect potential single cell variability. In recent years, live cell imaging has been employed to address heterogeneity at the single cell level [27], [28], [29]. We assessed the growth characteristics of *C. glutamicum* at the single cell level by means of live cell imaging. For this purpose microfluidic chambers were employed. These chambers are designed to hold cells at a single focal plane while supplying cells with a continuous flow of nutrients permitting monitoring of cell cycle events over multiple generations. To allow for accurate measurements and subsequently unambiguously define the cell poles and division septa, we made use of a DivIVA-mCherry expressing strain, where expression is under the control of the native promoter (Movie S1) [30]. In *C. glutamicum*, DivIVA localises at the cell poles and is recruited at a late stage to the mature division septum, which after completion of division forms the new cell pole [31].

**Figure 1:**
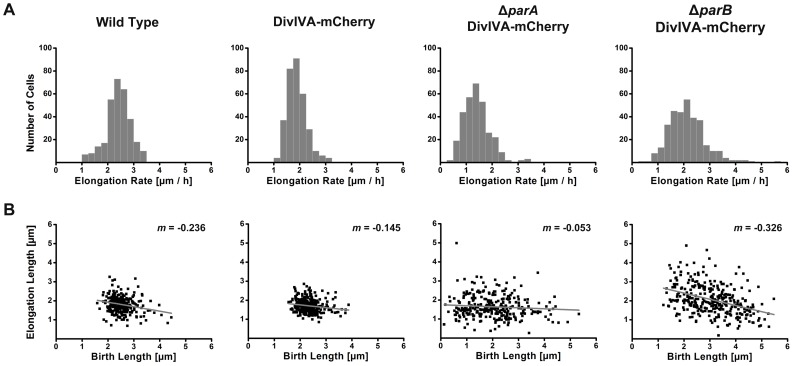
Chromosome segregation defects lead to altered growth in *C. glutamicum*. (**A**) Distribution of elongation rates of wild type, DivIVA-mCherry, Δ*parA* DivIVA-mCherry and Δ*parB* DivIVA-mCherry mutant cells. (*n* ≥300). (**B**) Association of birth lengths and elongation lengths in DivIVA-mCherry, Δ*parA* DivIVA-mCherry and Δ*parB* DivIVA-mCherry. In wild type and DivIVA-mCherry cells the birth length and elongation length are associated. In the absence of *parA* or *parB*, more variation in the length of the cell at birth and the cell length before division is observed. Negative correlation between birth and elongation lengths indicates a size-based cell cycle regulatory mechanism (grey line). Regression line slope is shown on top right. (*n* ≥300).

Growth of the DivIVA-mCherry expressing strain was assessed by measuring the increase in cell length per unit time directly after one division event (cell birth) until directly before the next division event. The elongation rate is in the range of 1 to 3.25 µm/h, averaging at 1.9 µm/h and follows a Gaussian distribution (Fig. 1A). Although cell division in *C. glutamicum* does not necessarily give rise to two equally sized daughter cells, the Gaussian distribution of elongation rates would suggest that the birth length, determined by the placement of the division septum, and the elongation rate are correlated. Indeed, plotting the birth length (size of cell directly after division) against the elongation length (the increase in cell length between two related division events) demonstrated that these two parameters are associated. The birth length of *C. glutamicum* cells ranges between 1.5 and 2.5 µm and these cells subsequently double in size (ranging from 1 to 2.5 µm) before the next division event takes place (Fig. 1A). Taken together, these data show that wild type *C. glutamicum* cells retain a relatively homogenous size both at birth and prior to division.

To assess the functionality of the DivIVA-mCherry fusion, the growth characteristics of wild type *C. glutamicum* was measured (Fig. S1 and Movie S2). Similar to the DivIVA-mCherry strain, the elongation rate of wild type cells follows a Gaussian distribution (Fig. 1A). The elongation rate ranges from 1 to 3.25 µm/h, however the average elongation rate of the wild type strain is increased compared to the DivIVA-mCherry strain (2.4 µm/h and 1.9 µm/h, respectively). The association of the birth length and the elongation length revealed a comparable association when compared to the DivIVA-mCherry strain (Fig. 1B). Considering that DivIVA is involved in a number of cellular processes in *C. glutamicum*, the results presented above show that the DivIVA-mCherry fusion is largely functional.

The relationship between birth length and elongation length has been used to broadly define the mechanism that regulates the timing of division events [28], [32]. If birth lengths negatively correlate with elongation length, a size-based regulatory mechanism is responsible. On the other hand, a time-based mechanism shows no correlation between birth length and elongation length. Using this approach it was recently demonstrated that *Mycobacterium* cells employ a time-based mechanism, while *E. coli* cells employ a size-based mechanism [28]. When assessed for wild type *C. glutamicum* or the DivIVA-mCherry strain, we found that birth lengths negatively correlate with elongation lengths, suggesting that a size-based or a mixed regulatory mechanism is employed (Fig. 1B).

### Chromosome Segregation Impacts on Cell Growth in *C. glutamicum*

Mutation of the *C. glutamicum* chromosome segregation machinery (*parA* or *parB*) induce a plethora of phenotypes, including altered growth rates and cell lengths, anucleate cells, guillotined chromosomes and perturbed chromosome organization [26]. These phenotypic consequences led us to speculate that chromosome segregation and/or organization might be coupled to division site selection and cell growth. With this in mind, the growth characteristics of *parA* and *parB* mutants were analyzed using live cell imaging (Fig. S1, Movies S3 and S4). Therefore, a divIVA-mCherry fusion was introduced into markerless *parA* or *parB* deletion background strains.

In cells lacking *parA* or *parB*, elongation rates varied from the DivIVA-mCherry strain (Fig. 1A, F<0.05). In the absence of *parA*, the elongation rate is reduced, while more variability is observed for the *parB* mutant, ranging from 0.25 µm/h to greater than 5 µm/h. Variation in the elongation rate might be due to deviating cell lengths at birth. Shorter cells at birth would require more time to reach a threshold size, while longer cells would require less time. Therefore, the association between birth length and elongation length was measured. In the absence of *parA* or *parB*, both birth lengths and elongation lengths are more variable (Fig 1B). However, birth and elongation lengths remain negatively correlated. Taken together, mutation of *parA* or *parB* not only alters chromosome organization, but also gives rise to a population of cells that is extremely heterogeneous both in birth size and elongation rate.

### Chromosome Segregation Influences Division Site Selection in *C. glutamicum*

The inconsistency of cell lengths at birth between wild type or DivIVA-mCherry and *par* mutant cells would suggest that the placement of the division septum is more variable in the absence of functional chromosome segregation machinery. As *C. glutamicum* lacks recognisable homologues of the conventional division site regulatory systems and no other positive or negative regulation of cell division are presently known, we speculated that chromosome segregation or the subcellular organization of the nucleoid might influence division site selection.

**Figure 2:**
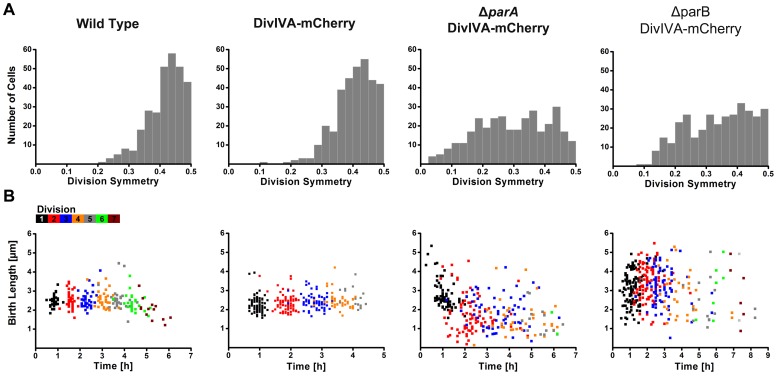
Chromosome segregation influences spatial and temporal control of cell division. (**A**) Distribution of division site placement in wild type, DivIVA-mCherry, Δ*parA* DivIVA-mCherry and Δ*parB* DivIVA-mCherry cells. In wild type and DivIVA-mCherry cells, division septa are placed near the midcell. In the absence of *parA* or *parB*, the positioning of division septa is more variable (F>0.05). (*n* ≥300). (**B**) Timing of birth events in wild type, Δ*parA* and Δ*parB* cells. In wild type and DivIVA-mCherry cells, division occurs at regular time intervals. Note that after 3–4 generations a microcolony is formed. In the absence of *parA* or *parB*, the time at which division occurs is random. In addition, the length of cells at birth is much more variable compared to wild type cells. (*n* ≥250).

On a single cell level, the placement of the division site in *C. glutamicum* cells was spatially (division symmetry) and temporally (timing of division events) assessed. Division symmetry was assessed by measuring the distance between the division septum and the nearest cell pole divided by the length of the cell, with 0.5 corresponding to the exact midcell. We found that the placement of the division site is not always precisely at midcell in wild type or DivIVA-mCherry cells; but some regulatory mechanism must exist because the division septum is sited in the region between the 1/3 and 2/3 positions of the cell (Fig. 2A). In the DivIVA-mCherry strain, we observed polar divisions occurring in a low percentage of the cells (0.3%) (Table 1). As DivIVA has been proposed to anchor the *oriC* at the cell poles through interaction with ParB, the low frequency of polar divisions is likely a consequence of the mCherry fusion which results in a mild chromosome segregation (anchoring) defect. However, in the absence of *parA* or *parB*, the placement of the division site is significantly more variable (Fig 2A; F<0.05). The frequency of polar cell division is drastically increased, often giving rise to nonviable, DNA-free cells (Table 1). Tracking division events over a number of generations revealed that divisions close to one cell pole often occurred repeatedly at the same pole. In almost every cell lineage analysed, in particular in the case of the *parA* mutant, asymmetric polar division was observed.

**Table 1:** Anucleate cells and polar divisions in wild type *C. glutamicum* and *par* mutants.

	Anucleate cells (*n* ≥650)	Polar divisions (*n* ≥300)
Wild Type	0%	0%
DivIVA-mCherry	0%	0.3%
Δ*parA* DivIVA-mCherry	18%	12%
Δ*parB* DivIVA-mCherry	28%	3.5%

In wild type and DivIVA-mCherry cells, the birth length and elongation length are correlated (Fig. 1B). Thus, starting from a single cell this would suggest that the timing of birth events would also be relatively synchronous. Indeed, at least for a number of generations, division events occur at regular intervals (Fig 2B). As outlined above, growth of the DivIVA-mCherry strain is reduced compared to the wild type (Fig. 1A). As a consequence, the timing of division events is delayed in the DivIVA-mCherry strain. Note that after 3 hours four division events take place in the wild type cells, while in the DivIVA-mCherry strain three division events are observed (Fig 2B). Nevertheless, in both strains division occurs at regular intervals. It should be pointed out that after some time a microcolony is formed, and in these microcolonies nutrient availability and growth are altered compared to single cells. In contrast, cells lacking *parA* or *parB* have more variable birth lengths and elongation lengths, and subsequently the timing of division events is random (Fig 2B). This result strongly indicates that alterations in chromosome localization, as a consequence of *parA* or *parB* mutation, directly influence cell growth and division site placement in *C. glutamicum*.

### Defects in Chromosome Segregation Lead to Chromosome Fragmentation

Next, we wanted to visualize the nucleoid localization and dynamics during live cell imaging, in particular in the absence of *parA* or *parB*. Cells were grown in the microfluidic chamber for a number of generations prior to staining cells with Hoechst DNA stain. As the DNA stain is somewhat toxic, growth of the cells is impaired. However, division events that have been initiated prior to addition of Hoechst stain are completed and hence, allow visualization of septum constriction over the chromosome.

**Figure 3:**
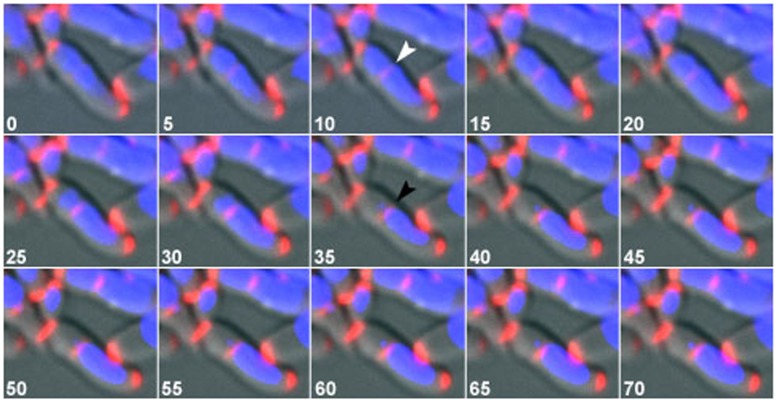
In the absence of *parB*, division septa constrict over the nucleoid, guillotining part of the chromosome. Shown is a time lapse series of DivIVA-mCherry expressing cell in the absence of *parB*. Images were acquired every 5 minutes and time points are indicated for each time frame. DNA is stained with Hoechst (blue) and DivIVA-mCherry is shown in red. DivIVA-mCherry is recruited to the division septum, which has assembled over the chromosome (white arrowhead). The chromosome is pumped in one direction, into the cell half containing the bulk of the chromosome. The division septum constricts directly over part of the chromosome, leading to guillotining part of the chromosome (black arrowhead). See also Movie S5.

In wild type and DivIVA-mCherry cells, each daughter cell always contained a nucleoid. However, in the *parA* or *parB* mutant strains, a high frequency of anucleate cells were observed (Table 1). Additionally, constriction of the division septum over the nucleoid was observed. In the case of Δ*parA* 9% of the division septa constricted over the chromosome, while in the case of Δ*parB* 10.5% was observed (*n* ≥650). In the example shown (Fig. 3 and Movie S5), the division septum is positioned over the nucleoid and as the septum begins constricting the DNA is pumped into the cell half containing the bulk of the chromosome. DNA translocases, such as FtsK in *E. coli*, coordinate the late stages of cytokinesis and chromosome segregation ensuring that DNA does not get trapped in the inward growing septum [33], [34]. *C. glutamicum* contains a homolog of FtsK, however it has not been studied in detail, to date. Nevertheless, in the example shown DNA translocase did not succeed in clearing the DNA from the invaginating septum. Subsequently, part of the chromosome was fragmented and then degraded. Our results confirm earlier postulations [35] that *C. glutamicum* likely lacks a nucleoid occlusion protein, such as SlmA or Noc.

## Discussion

Most of our knowledge on bacterial growth and cell division has been derived from the rod-shaped model organisms such as *B. subtilis* and *E. coli*. However, many of the symmetry generating molecular rulers are lacking in other rod-shaped bacteria, for example the rod-shaped actinomycete *C. glutamicum*. Here, growth and division site selection in *C. glutamicum* was analyzed at the single cell level.

Cell elongation in many rod-shaped bacteria involves intercalation of new peptidoglycan into the lateral cell wall [36], [37], [38]. The actin cytoskeleton is not only imperative to this mode of growth but also to the maintenance of rod-shape [39], [40], [41], [42]. By contrast, Actinobacteria, which lack an MreB-based cell elongation machinery, insert new cell wall material at the cell poles, giving rise to an apical mode of cell elongation [25], [31], [43]. Apical growth in Actinomycetes has been reported to be organized by DivIVA [31], [43], [44]. In this study, the localization pattern of DivIVA was exploited to mark the cell poles and constricting division septa in growing *C. glutamicum* cells.

*E. coli* and *B. subtilis* cells normally exhibit little size variation under steady state conditions [45], [46]. Heterogeneity in cell size could arise from alterations in the placement of the division septum. Thus, regulating the placement of the division site additionally helps maintain a homogenous cell size. By contrast, asymmetry in division site selection as well as polar cell growth was shown for mycobacterial cells [28]. Such asymmetry results in an extremely heterogeneous population of cells with diverse susceptibility to antibiotics. In *C. glutamicum*, cell division does not always give rise to equally sized daughter cells (Fig. 2A). Although the majority of cells divide close to the midcell region, the fraction of cell dividing off-centre could lead to cell size heterogeneity. Interestingly, we find that the growth rate of *C. glutamicum* cells follows a Gaussian distribution, ranging between 1–3.5 µm/h (Fig. 2A). To gain additional information on the homo- or heterogeneity of the population, the association of birth lengths and elongation lengths was assessed. Good correlation between birth and elongation lengths was observed, suggesting that *C. glutamicum* maintains a relatively homogenous cell size both prior to and after division (Fig. 1B). Variability in division site placement must be compensated for by increased or decreased growth rate, maintaining a threshold cell size before the next division event occurs. This finding is in line with the observation that birth lengths negatively correlate with elongation length, suggesting that *C. glutamicum* employs a size-based regulatory mechanism. However, we also observe that division events occur with relative precise timing in wild type *C. glutamicum* cells. This would suggest that *C. glutamicum* employs a mixture of time- and size-based regulatory mechanisms. This is an interesting difference to growth control to the closely related *Mycobacteria*. For *M. smegmatis* and *M. tuberculosis* only a time-based mechanism was proposed [28]. A major difference in the lifestyle between *Corynebacteria* and *Mycobacteria* is that *Corynebacteria* grow with much higher growth rates. Thus, it seems plausible that these cells not only rely on one control mechanism, but rather follow a mixed strategy to avoid that cells where division failed will grow out to large filaments.

In the absence of an apparent Min system and a clear Noc/SlmA homologue, how is division site regulated in *C. glutamicum*? We previously reported that mutation of the *parAB* partition system resulted in altered cell length distributions, aberrant placement of the division septa and a high frequency of anuncleate cells [26]. When analyzed at the single cell level, we observed much more variability in division site selection, high frequency of polar divisions, along with significantly more variable birth lengths that do not correlate with elongation length. Although we do not know the molecular mechanism(s) that regulate growth and division site selection in *C. glutamicum*, we speculate that the nucleoid itself or some aspect associated with the nucleoid influences these parameters.

From the data presented here it appears that cell division and chromosome segregation are coupled in *C. glutamicum*. In *B. subtilis*, blocking cell division by overexpression of the Min system results in long filamentous cells with regularly spaced and segregated chromosomes. Conversely, mutation of *spo0J* (*parB*) or *soj* (*parA*) do not lead to severe cell division defects, approximately 2% anucleate cells result from mutation of *spo0J* [47]. Thus, cell division and chromosome segregation can be uncoupled in *B. subtilis*, probably as a consequence of partially overlapping cell cycle regulatory mechanisms.

A growing body of evidence suggests that the nucleoid morphology directly influences Z-ring positioning [22], [24], [48], [49], [50], [51], [52]. Blocking DNA replication at different stages induces different nucleoid morphologies which influence Z-ring positioning [49]. Thus, even in *B. subtilis*, *E. coli* and *C. crescentus* additional factors, potentially related to chromosome replication or the nucleoid structure, prime the midcell for Z-ring polymerization [48]. In line with these observations, alterations in the nucleoid structure lead to aberrant placement of the division site, in *C. glutamicum*.

## Materials and Methods

### Bacterial Strains

Bacterial strains and plasmids used in this study are listed in table 2.

**Table 2:** Bacterial strains and plasmids.

**Strains**	**Relevant characteristics**	**Reference/source**
***C. glutamicum*** ** RES 167**	Restriction deficient *C. glutamicum* mutant, otherwise considered wild type	[53]
**CDC010**	RES 167 derivative with *divIVA*-mCherry	[30]
**CDC025**	RES 167 Δ*parA*, *divIVA*-mCherry	This study
**CDC012**	RES 167 Δ*parB*, *divIVA*-mCherry	[30]
**Plasmids**	**Relevant characteristics**	**Reference/source**
**pK19mobsacB**	Integration vector, *ori* pUC, Km^r^, *mob sacB*	[54]
**pK19mobsacB-ΔparA**	Integration vector, *ori* pUC, Km^r^, *mob sacB,* Δ*parA*	[26]
**pK19mobsacB-ΔparB**	Integration vector, *ori* pUC, Km^r^, *mob sacB,* Δ*parB*	[26]
**pCD191**	Integration vector, *ori* pUC, Km^r^, *mob sacB divIVA-mCHERRY*	[26]

Strain CDC025 was generated by transforming strain CDC001 (Δ*parA*) with the plasmid pCD191 (DivIVA-mCherry) via electroporation. This strain contains an in-frame deletion of *parA* and expression of a DivIVA-mCherry fusion from the native promoter. Plasmid pCD191 is a pK19mob*sacB* derivative, which is nonreplicative in *C. glutamicum* [26], [54]. Chromosomal integration of the *mCherry* gene at the 3' end of the *divIVA* locus occurs via a two-step homologous recombination. The initial chromosome integration step was selected for on kanamycin plates. The second round of recombination was selected for by growth on 10% sucrose. Single colonies were isolated and tested for kanamycin sensitivity. Chromosomal integration of *mCherry* was confirmed by PCR.

### Time-lapse Microscopy with Microfluidic Chambers

Live cell imaging was carried out in B04A microfluidic chamber (Onix, CellASIC). *C. glutamicum* cells were grown in BHI medium overnight. The next morning, cultures were diluted to OD_600_ 1.0 and grown further in shaking flasks to approximately OD_600_ 5.0. Cultures were diluted to OD_600_ 0.005–0.01 prior to loading microfluidic chamber. Cells were loaded to wells with 5 psi for 10 seconds. Nutrient supply was maintained at 3 psi. The temperature was maintained at 30°C. Cells were grown in BHI during time lapse analysis and images were acquired at five minute intervals. For anucleate cell measurements and analysis of constricting division septa over the chromosome, cells were stained with Hoechst DNA stain (1 µg/ml^−1^). Images were taken on a Delta Vision RT microscope (Applied Precision), using the sofworX software. Final image preparation was done using Volocity, Adobe Photoshop 6.0 (Adobe Systems Incorporated) or FIJI.

### Statistical Analysis

All cell length measurements were acquired manually. Two-sample for variances F-test were calculated using Excel to determine the variability in distribution between wild type/DivIVA-mCherry and Δ*parA* or Δ*parB*.

## Supporting Information

Figure S1 **Still images of DivIVA-mCherry and** ***par*** **mutants grown in microfluidic chambers.** Shown are still images of (A) DivIVA-mCherry, (B) Δ*parB* DivIVA-mCHERRY and (C) Δ*parA* DivIVA-mCHERRY. The arrows in (C) show a cell where the division septum is positioned close to the cell pole. In the same cell, the division septum in the following division event is also positioned close to the same cell pole. Time points are indicated in minutes on the top left corner.
(TIF)

Movie S1 **Growth of** ***C. glutamicum*** **DivIVA-mCHERRY cells.** Images were acquired every five minutes for the total of 5 hours.
(AVI)

Movie S2 **Growth of wild type** ***C. glutamicum*** **cells.** Images were acquired every five minutes for the total of 5 hours.
(M4V)

Movie S3 **Growth of** ***C. glutamicum*** **Δ** ***parA*** **DivIVA-mCHERRY cells.** Images were acquired every 5 minutes for a total of 7 hours.
(AVI)

Movie S4 **Growth of** ***C. glutamicum*** **Δ** ***parB*** **DivIVA-mCHERRY cells.** Images were acquired every 5 minutes for a total of 3 hours 40 minutes.
(AVI)

Movie S5 **In cells lacking** ***parB*** **, the division septum guillotines part of the chromosome.**
(AVI)

## References

[1] Bi EF, Lutkenhaus J (1991) FtsZ ring structure associated with division in *Escherichia coli* . Nature 354: 161–164. 1944597 10.1038/354161a0

[2] Wang X, Lutkenhaus J (1993) The FtsZ protein of *Bacillus subtilis* is localized at the division site and has GTPase activity that is dependent upon FtsZ concentration. Mol Microbiol 9: 435–442. 8412693 10.1111/j.1365-2958.1993.tb01705.x

[3] Bramkamp M, van Baarle S (2009) Division site selection in rod-shaped bacteria. Curr Opin Microbiol 12: 683–688. 19884039 10.1016/j.mib.2009.10.002

[4] Bernhardt TG, de Boer PA (2005) SlmA, a nucleoid-associated, FtsZ binding protein required for blocking septal ring assembly over Chromosomes in *E. coli* . Mol Cell 18: 555–564. 15916962 10.1016/j.molcel.2005.04.012 PMC4428309

[5] Wu LJ, Ishikawa S, Kawai Y, Oshima T, Ogasawara N, et al (2009) Noc protein binds to specific DNA sequences to coordinate cell division with chromosome segregation. Embo J 28: 1940–1952. 19494834 10.1038/emboj.2009.144 PMC2711181

[6] Wu LJ, Errington J (2004) Coordination of cell division and chromosome segregation by a nucleoid occlusion protein in *Bacillus subtilis* . Cell 117: 915–925. 15210112 10.1016/j.cell.2004.06.002

[7] de Boer PA, Crossley RE, Rothfield LI (1988) Isolation and properties of *minB*, a complex genetic locus involved in correct placement of the division site in *Escherichia coli.* . J Bacteriol 170: 2106–2112. 2834323 10.1128/jb.170.5.2106-2112.1988 PMC211093

[8] de Boer PA, Crossley RE, Rothfield LI (1989) A division inhibitor and a topological specificity factor coded for by the minicell locus determine proper placement of the division septum in *E. coli* . Cell 56: 641–649. 2645057 10.1016/0092-8674(89)90586-2

[9] Hu Z, Lutkenhaus J (1999) Topological regulation of cell division in *Escherichia coli* involves rapid pole to pole oscillation of the division inhibitor MinC under the control of MinD and MinE. Mol Microbiol 34: 82–90. 10540287 10.1046/j.1365-2958.1999.01575.x

[10] Raskin DM, de Boer PA (1999) MinDE-dependent pole-to-pole oscillation of division inhibitor MinC in *Escherichia coli* . J Bacteriol 181: 6419–6424. 10515933 10.1128/jb.181.20.6419-6424.1999 PMC103778

[11] Hale CA, Meinhardt H, de Boer PA (2001) Dynamic localization cycle of the cell division regulator MinE in *Escherichia coli* . Embo J 20: 1563–1572. 11285221 10.1093/emboj/20.7.1563 PMC145461

[12] Lutkenhaus J (2002) Dynamic proteins in bacteria. Curr Opin Microbiol 5: 548–552. 12457696 10.1016/s1369-5274(02)00376-4

[13] van Baarle S, Bramkamp M (2010) The MinCDJ system in *Bacillus subtilis* prevents minicell formation by promoting divisome disassembly. PLoS One 5: e9850. 20352045 10.1371/journal.pone.0009850 PMC2844427

[14] Gregory JA, Becker EC, Pogliano K (2008) *Bacillus subtilis* MinC destabilizes FtsZ-rings at new cell poles and contributes to the timing of cell division. Genes Dev 22: 3475–3488. 19141479 10.1101/gad.1732408 PMC2607072

[15] Hayes F, Barilla D (2006) The bacterial segrosome: a dynamic nucleoprotein machine for DNA trafficking and segregation. Nat Rev Microbiol 4: 133–143. 16415929 10.1038/nrmicro1342

[16] Fogel MA, Waldor MK (2006) A dynamic, mitotic-like mechanism for bacterial chromosome segregation. Genes Dev 20: 3269–3282. 17158745 10.1101/gad.1496506 PMC1686604

[17] Leonard TA, Møller-Jensen J, Löwe J (2005) Towards understanding the molecular basis of bacterial DNA segregation. Philos Trans R Soc Lond B Biol Sci 360: 523–535. 15897178 10.1098/rstb.2004.1608 PMC1569471

[18] Funnell BE (1988) Mini-P1 plasmid partitioning: excess ParB protein destabilizes plasmids containing the centromere *parS* . J Bacteriol 170: 954–960. 2962991 10.1128/jb.170.2.954-960.1988 PMC210747

[19] Ptacin JL, Lee SF, Garner EC, Toro E, Eckart M, et al (2010) A spindle-like apparatus guides bacterial chromosome segregation. Nat Cell Biol 12: 791–798. 20657594 10.1038/ncb2083 PMC3205914

[20] Collier J (2012) Regulation of chromosomal replication in *Caulobacter crescentus* . Plasmid 67: 76–87. 22227374 10.1016/j.plasmid.2011.12.007

[21] Toro E, Shapiro L (2010) Bacterial chromosome organization and segregation. Cold Spring Harb Perspect Biol 2: a000349. 20182613 10.1101/cshperspect.a000349 PMC2828278

[22] Thanbichler M, Shapiro L (2006) MipZ, a spatial regulator coordinating chromosome segregation with cell division in *Caulobacter* . Cell 126: 147–162. 16839883 10.1016/j.cell.2006.05.038

[23] Kiekebusch D, Michie KA, Essen LO, Löwe J, Thanbichler M (2012) Localized dimerization and nucleoid binding drive gradient formation by the bacterial cell division inhibitor MipZ. Mol Cell 46: 245–259. 22483621 10.1016/j.molcel.2012.03.004 PMC3355305

[24] Mohl DA, Gober JW (1997) Cell cycle-dependent polar localization of chromosome partitioning proteins in *Caulobacter crescentus* . Cell 88: 675–684. 9054507 10.1016/s0092-8674(00)81910-8

[25] Letek M, Fiuza M, Ordonez E, Villadangos AF, Ramos A, et al (2008) Cell growth and cell division in the rod-shaped actinomycete *Corynebacterium glutamicum* . Antonie Van Leeuwenhoek 94: 99–109. 18283557 10.1007/s10482-008-9224-4

[26] Donovan C, Schwaiger A, Krämer R, Bramkamp M (2010) Subcellular localization and characterization of the ParAB system from *Corynebacterium glutamicum* . J Bacteriol 192: 3441–3451. 20435732 10.1128/JB.00214-10 PMC2897671

[27] Siegal-Gaskins D, Crosson S (2008) Tightly regulated and heritable division control in single bacterial cells. Biophys J 95: 2063–2072. 18469083 10.1529/biophysj.108.128785 PMC2483777

[28] Aldridge BB, Fernandez-Suarez M, Heller D, Ambravaneswaran V, Irimia D, et al (2012) Asymmetry and aging of mycobacterial cells lead to variable growth and antibiotic susceptibility. Science 335: 100–104. 22174129 10.1126/science.1216166 PMC3397429

[29] Megerle JA, Fritz G, Gerland U, Jung K, Rädler JO (2008) Timing and dynamics of single cell gene expression in the arabinose utilization system. Biophys J 95: 2103–2115. 18469087 10.1529/biophysj.107.127191 PMC2483754

[30] Donovan C, Sieger B, Krämer R, Bramkamp M (2012) A synthetic *Escherichia coli* system identifies a conserved origin tethering factor in Actinobacteria. Mol Microbiol 84: 105–116. 22340668 10.1111/j.1365-2958.2012.08011.x

[31] Letek M, Ordonez E, Vaquera J, Margolin W, Flärdh K, et al (2008) DivIVA is required for polar growth in the MreB-lacking rod-shaped actinomycete *Corynebacterium glutamicum* . J Bacteriol 190: 3283–3292. 18296522 10.1128/JB.01934-07 PMC2347398

[32] Sveiczer A, Novak B, Mitchison JM (1996) The size control of fission yeast revisited. J Cell Sci 109 (Pt 12): 2947–2957. 10.1242/jcs.109.12.2947 9013342

[33] Bigot S, Sivanathan V, Possoz C, Barre FX, Cornet F (2007) FtsK, a literate chromosome segregation machine. Mol Microbiol 64: 1434–1441. 17511809 10.1111/j.1365-2958.2007.05755.x

[34] Lesterlin C, Barre FX, Cornet F (2004) Genetic recombination and the cell cycle: what we have learned from chromosome dimers. Mol Microbiol 54: 1151–1160. 15554958 10.1111/j.1365-2958.2004.04356.x

[35] Ramos A, Letek M, Campelo AB, Vaquera J, Mateos LM, et al (2005) Altered morphology produced by *ftsZ* expression in *Corynebacterium glutamicum* ATCC 13869. Microbiology 151: 2563–2572. 16079335 10.1099/mic.0.28036-0

[36] Wachi M, Doi M, Tamaki S, Park W, Nakajima-Iijima S, et al (1987) Mutant isolation and molecular cloning of *mre* genes, which determine cell shape, sensitivity to mecillinam, and amount of penicillin-binding proteins in *Escherichia coli* . J Bacteriol 169: 4935–4940. 2822655 10.1128/jb.169.11.4935-4940.1987 PMC213889

[37] Jones LJ, Carballido-Lopez R, Errington J (2001) Control of cell shape in bacteria: helical, actin-like filaments in *Bacillus subtilis* . Cell 104: 913–922. 11290328 10.1016/s0092-8674(01)00287-2

[38] Daniel RA, Errington J (2003) Control of cell morphogenesis in bacteria: two distinct ways to make a rod-shaped cell. Cell 113: 767–776. 12809607 10.1016/s0092-8674(03)00421-5

[39] Carballido-Lopez R (2006) The bacterial actin-like cytoskeleton. Microbiol Mol Biol Rev 70: 888–909. 17158703 10.1128/MMBR.00014-06 PMC1698507

[40] Chastanet A, Carballido-Lopez R (2012) The actin-like MreB proteins in *Bacillus subtilis*: a new turn. Front Biosci (Schol Ed) 4: 1582–1606. 22652894 10.2741/s354

[41] Dominguez-Escobar J, Chastanet A, Crevenna AH, Fromion V, Wedlich-Söldner R, et al (2011) Processive movement of MreB-associated cell wall biosynthetic complexes in bacteria. Science 333: 225–228. 21636744 10.1126/science.1203466

[42] Garner EC, Bernard R, Wang W, Zhuang X, Rudner DZ, et al (2011) Coupled, circumferential motions of the cell wall synthesis machinery and MreB filaments in *B. subtilis* . Science 333: 222–225. 21636745 10.1126/science.1203285 PMC3235694

[43] Flärdh K (2003) Essential role of DivIVA in polar growth and morphogenesis in *Streptomyces coelicolor* A3(2). Mol Microbiol 49: 1523–1536. 12950918 10.1046/j.1365-2958.2003.03660.x

[44] Kang CM, Nyayapathy S, Lee JY, Suh JW, Husson RN (2008) Wag31, a homologue of the cell division protein DivIVA, regulates growth, morphology and polar cell wall synthesis in mycobacteria. Microbiology 154: 725–735. 18310019 10.1099/mic.0.2007/014076-0

[45] Weart RB, Lee AH, Chien AC, Haeusser DP, Hill NS, et al (2007) A metabolic sensor governing cell size in bacteria. Cell 130: 335–347. 17662947 10.1016/j.cell.2007.05.043 PMC1971218

[46] Grover NB, Woldringh CL (2001) Dimensional regulation of cell-cycle events in *Escherichia coli* during steady-state growth. Microbiology 147: 171–181. 11160811 10.1099/00221287-147-1-171

[47] Ireton K, Gunther NW, Grossman AD (1994) Spo0J is required for normal chromosome segregation as well as the initiation of sporulation in *Bacillus subtilis* . J Bacteriol 176: 5320–5329. 8071208 10.1128/jb.176.17.5320-5329.1994 PMC196717

[48] Rodrigues CD, Harry EJ (2012) The Min system and nucleoid occlusion are not required for identifying the division site in *Bacillus subtilis* but ensure its efficient utilization. PLoS Genet 8: e1002561. 22457634 10.1371/journal.pgen.1002561 PMC3310732

[49] Moriya S, Rashid RA, Rodrigues CD, Harry EJ (2010) Influence of the nucleoid and the early stages of DNA replication on positioning the division site in *Bacillus subtilis* . Mol Microbiol 76: 634–647. 20199598 10.1111/j.1365-2958.2010.07102.x

[50] Harry E, Monahan L, Thompson L (2006) Bacterial cell division: the mechanism and its precison. Int Rev Cytol 253: 27–94. 17098054 10.1016/S0074-7696(06)53002-5

[51] Migocki MD, Freeman MK, Wake RG, Harry EJ (2002) The Min system is not required for precise placement of the midcell Z ring in *Bacillus subtilis* . EMBO Rep 3: 1163–1167. 12446561 10.1093/embo-reports/kvf233 PMC1308329

[52] Mohl DA, Easter J Jr, Gober JW (2001) The chromosome partitioning protein, ParB, is required for cytokinesis in *Caulobacter crescentus* . Mol Microbiol 42: 741–755. 11722739 10.1046/j.1365-2958.2001.02643.x

[53] Tauch A, Kirchner O, Loffler B, Gotker S, Pühler A, et al (2002) Efficient electrotransformation of *Corynebacterium diphtheriae* with a mini-replicon derived from the *Corynebacterium glutamicum* plasmid pGA1. Curr Microbiol 45: 362–367. 12232668 10.1007/s00284-002-3728-3

[54] Schäfer A, Tauch A, Jäger W, Kalinowski J, Thierbach G, et al (1994) Small mobilizable multi-purpose cloning vectors derived from the *Escherichia coli* plasmids pK18 and pK19: selection of defined deletions in the chromosome of *Corynebacterium glutamicum* . Gene 145: 69–73. 8045426 10.1016/0378-1119(94)90324-7

