# Supplementary figures and images for: Chromosome Segregation Impacts on Cell Growth and Division Site Selection in Corynebacterium glutamicum
Source: PLoS One. 2013 Feb 6;8(2):e55078. doi: 10.1371/journal.pone.0055078 (PMC3566199; doi:10.1371/journal.pone.0055078)

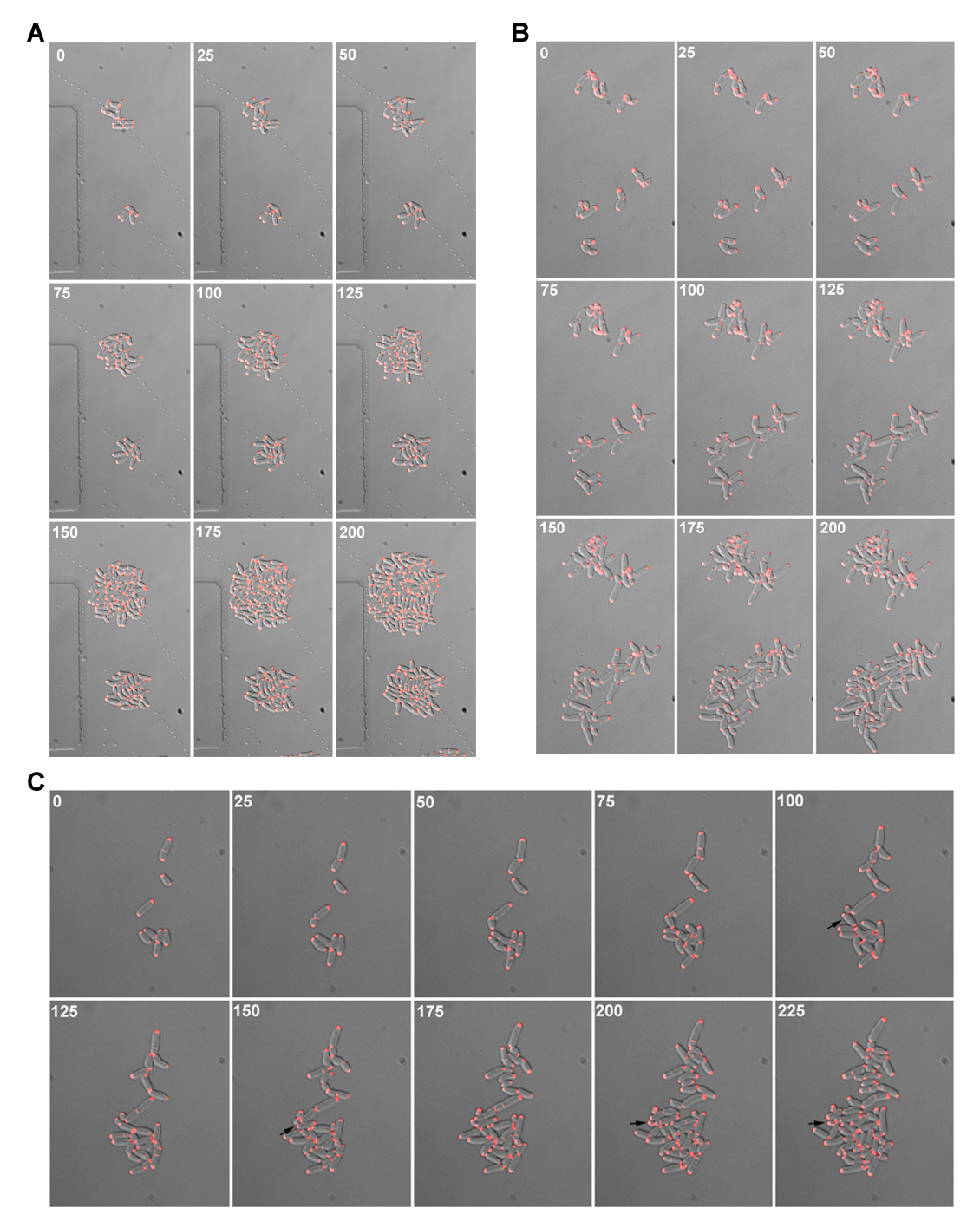

Supplement: Figure S1 — Still images of DivIVA-mCherry and par mutants grown in microfluidic chambers. Shown are still images of (A) DivIVA-mCherry, (B) ΔparB DivIVA-mCHERRY and (C) ΔparA DivIVA-mCHERRY. The arrows in (C) show a cell where the division septum is positioned close to the cell pole. In the same cell, the division septum in the following division event is also positioned close to the same cell pole. Time points are indicated in minutes on the top left corner. (TIF) [file pone.0055078.s001.tif]
